# Highlight selection of radiochemistry and radiopharmacy developments by editorial board

**DOI:** 10.1186/s41181-023-00192-5

**Published:** 2023-03-23

**Authors:** Oliver C. Kiss, Peter J. H. Scott, Martin Behe, Ivan Penuelas, Jan Passchier, Ana Rey, Marianne Patt, Silvio Aime, Amir Jalilian, Peter Laverman, Zhen Cheng, Alain Faivre Chauvet, Jonathan Engle, Frederik Cleeren, Hua Zhu, Johnny Vercouillie, Michael van Dam, Ming Rong Zhang, Lars Perk, Benjamin Guillet, Francisco Alves

**Affiliations:** 1 Helmholtz Zentrum Dresden Rossendorf, Dresden, Germany; 2 University of Michigan, Ann Arbor, MI USA; 3 Paul Scherrer Institute, Villigen, Switzerland; 4 University Clinic of Navarra, Pamplona, Spain; 5 Invicro, Imperial College London, London, UK; 6 Universidad de la Rebublica, Montevideo, Uruguay; 7 University of Leipzig, Leipzig, Germany; 8 University of Torino, Turin, Italy; 9 IAEA, Vienna, Austria; 10 Radboud University Medical Center, Nijmegen, The Netherlands; 11 Shanghai Institute of Materia Medica, Shanghai, China; 12 Inserm, Nantes, France; 13 University of Wisconsin, Madison, WI USA; 14 Katholieke Universiteit, Leuven, Belgium; 15 Peking University Cancer Hospital, Beijing, China; 16 University of Tours, Tours, France; 17 UCLA, Los Angeles, USA; 18 NIRS, Chiba, Japan; 19 CERIMED, Marseille, France; 20 University of Coimbra, Coimbra, Portugal

**Keywords:** Highlight articles, Radiochemistry, Radiopharmacy, Radiopharmaceutical Sciences, Nuclear medicine, Trends in radiopharmaceutical sciences

## Abstract

**Background:**

The Editorial Board of EJNMMI Radiopharmacy and Chemistry releases a biannual highlight commentary to update the readership on trends in the field of radiopharmaceutical development.

**Main Body:**

This selection of highlights provides commentary on 21 different topics selected by each coauthoring Editorial Board member addressing a variety of aspects ranging from novel radiochemistry to first-in-human application of novel radiopharmaceuticals.

**Conclusion:**

Trends in radiochemistry and radiopharmacy are highlighted. Hot topics cover the entire scope of EJNMMI Radiopharmacy and Chemistry, demonstrating the progress in the research field, and include new PET-labelling methods for ^11^C and ^18^F, the importance of choosing the proper chelator for a given radioactive metal ion, implications of total body PET on use of radiopharmaceuticals, legislation issues and radionuclide therapy including the emerging role of ^161^Tb.

## Background

Each individual coauthoring member of the Editorial Board has selected to highlight an article that has appeared in the radiochemistry, radiopharmacy and imaging agent literature during the period July-December 2022. The aim of this collaborative initiative is to create a biyearly overview for the readers summarizing the latest trends and hot topics in the field.

## Main text

### Production of ^11^C-labeled α-amino acids by reaction of unlabeled α-amino acids with [^11^C]CO_2_

#### By Ming-Rong Zhang

^11^C-Labeled α-amino acids are widely used for PET imaging of cancer. Because of the short half-life of ^11^C, the radiosynthesis of ^11^C-labeled amino acid remains challenging. The use of [^11^C]HCN for cyanation of aldehydes or ketones, followed by hydrolysis, has been a useful method for synthesizing ^11^C-labeled amino acids. However, the efficient production of [^11^C]HCN is challenging. Moreover, automated production of ^11^C-labeled amino acids requires complicated sequences and is time-consuming.

**Fig. 1:**
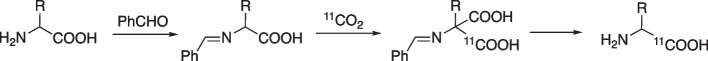
Synthetic scheme for synthesis of ^11^C-amino acids through isotopic exchange catalyzed by benzaldehyde

A new study reports that aldehydes catalyze the isotopic carboxylate exchange of native α-amino acids with cyclotron-produced [^11^C]CO_2_ (Bsharat et al. 2022). Proteinogenic α-amino acids and many non-natural variants containing different chemical functional groups can undergo efficient labelling. The reaction mechanism is considered as follows: the trapping of [^11^C]CO_2_ and reaction with unlabeled amino acids by imine-carboxylate intermediates to yield [^11^C]iminomalonates that are easy for [^11^C]monodecarboxylation (Fig. 1). Tempering catalyst electrophilicity was key to prevent irreversible aldehyde consumption. The pre-generation of the imine carboxylate intermediate allows for the rapid and late-stage ^11^C-radiolabelling of α-amino acids in the presence of [^11^C]CO_2_, without changing the chemical structures of α-amino acids.

Using this method, the authors synthesized [^11^C]tyrosine, [^11^C]tryptophan, [^11^C]leucine, [^11^C]glutamine, [^11^C]thyroxine, and [^11^C]glutathione. Moreover, they synthesized some ^14^C- and ^13^C-labeled α-amino acids with *CO_2_.

### Single domain engineered antibodies: are they really so good for theranostics?

#### By Iván Peñuelas

In the field of theranostics, immunoPET and immunoSPECT imaging have quickly evolved in the last decade. Despite their very many advantages (e.g. selectivity for their target), radiolabelled antibodies show slow clearance, non-optimal tumor-to-background ratios and long biodistribution times, all hindering their routine clinical use.

Being much smaller proteins, single domain antibodies (sdAb) present very fast clearance times and high penetration in tumors. sdAbs are easily engineerable, can be much more convenient for radiolabelling, and permit faster development of potential theranostic pairs, fostering translation to the clinic (Wei et al. 2022). sdAb can be obtained from human Ab repertoires, or isolated/engineered from camelids or sharks. These small proteins are much more resistant to harsh conditions than antibodies and can withstand high temperatures and quite extreme pH conditions, thus facilitating the development and use of many different radiolabelling strategies.

The fast biodistribution times of sdAb permits the use of widely available short-lived radionuclides such as technetium-99m and, more interestingly for full quantitation, PET radionuclides such as gallium-68 or even click chemistry approaches using fluorine-18. The panoply of radionuclides used (and in many cases transferred to the clinic in pilot studies) includes zirconium-89, gallium-68, fluorine-18, copper-64, technetium-99m, iodine-131, and indium-111. Several theranostic pairs (mainly using lutetium-177 as the therapeutic radionuclide) have also been used, and even alpha particle-labelled sdAbs have already been reported.

However, further research is needed to circumvent the disadvantages and downsides of the use of sdAb. In such small proteins (down to roughly 15 kDa in the case on nanobodies), their high renal retention could lead to significant nephrotoxicity when therapeutic radionuclides are used. The presence of the His_6_ tag, commonly used for engineered protein purification, increases renal retention, although replacement with the HEHEHE sequence has been shown to reduce it for affibody constructs. In addition, the use of pegylated sdAbs, administration to the patient of gelofusine, or co-administration of positively charged amino acid cocktails can reduce potential nephrotoxicity.

As a whole, sdAb seem to be very interesting “druggable” constructs for theranostic applications of radiolabelled immunocomplexes, and their use will probably increase in the near future for a wider range of applications.

### Choose your chelator wisely

#### By Peter Laverman

^89^Zr-labeled antibodies have emerged as a new class of radiopharmaceuticals since the first publications in the early 2000s (Verel et al. 2003; Vosjan et al. 2010). Since then, numerous preclinical and clinical studies have been described using either the TFP-*N*-suc-DFO-Fe-ester (DFN-*N*-suc) (Verel et al. 2003) or DFO-NCS (Vosjan et al. 2010). The development of DFO-NCS was a step forward due to its more straightforward use (no need for Fe-protection), but both chelators are not optimally stable and in vivo studies in mice revealed loss of ^89^Zr from the chelator in vivo. In search for a more stable chelator, DFO* (amongst others) was developed and compared to DFO (Vugts et al. 2017) as well as DFO and DFOcyclo* (Raavé et al. 2019). Traditionally, antibodies are known to be able to poorly cross the blood-brain barrier (BBB). One way to overcome this limitation is the use of transferrin as a vehicle to transport antibodies across the BBB. Recently, several amyloid-beta monoclonal antibodies have been modified with the scFv variant of an antibody targeting transferrin, which is expected to lead to enhanced brain uptake. To further elucidate the targeting of these antibodies, the ex vivo and in vivo behavior of the bispecific anti-amyloid-beta aducanumab derivate mAbAdu-scFab8D3 (Adu-8D3), labeled with either ^89^Zr via DFO-NCS, DFO-*N*-suc or DFO*-NCS, was evaluated in an APP/PS1 mouse model of Alzheimer’s Disease (Wuensche et al. 2022). The ^89^Zr-labeled antibodies were compared with the non-residualizing, directly-labeled, ^125^I-labeled antibodies. Interestingly, the specific brain uptake of the [^89^Zr]Zr-DFO*-labeled and ^125^I-labeled Adu-8D3 was significantly higher than that of the DFO-NCS and DFO-N-suc variants, which displayed only low and non-specific brain uptake. The reason for these differences is not fully understood, but the instability of the DFO-NCS and DFO-*N*-suc chelators may play a role, as well as the elevated occurrence of free iron species in the APP/PS1 TG mice, resulting in a competition of Fe^3+^ with Zr^4+^ for DFO*/DFO.

In conclusion, this paper further demonstrates the need for careful selection of the chelator for radiometal labeling, and also nicely shows the application of immunoPET in neurological applications.

### Carbon-11, an old wine in new bottles

#### By Oliver Kiss

Carbon-11 was discovered in 1934 and, since the emergence of PET scanners in the 70s, has been widely used in Nuclear Medicine. One of the advantages of carbon-11 lies in the presence of the natural occurence of carbon in almost all biologically active molecules, and therefore toxicological profiles are well known. Endogenous and natural exogenous compounds have been radiolabelled in the last few decades and evaluated in preclinical and clinical studies. In a recent review (Shegani et al. 2023), carbon-11 radiolabelled compounds have been divided into different categories i.e. alcohols, alkaloids, amino acids, enzyme cofactors and vitamines, endogenous gases, fatty acids, hormones and neurotransmitters, nucleotides, peptides, sugars and miscellaneous compounds.

For each category, examples of radiosyntheses are graphically depicted, and both preclinical and clinical results are illustrated giving insights into almost a century of research and clinical application of one of the most interesting radionuclides in our field. With the advent of whole-body and large field-of-view PET scanners and their benefits (*vide infra*), there is likely a renaissance of carbon-11 labelled radiopharmaceuticals (de Vries et al. 2022).

### Terbium-161 labeled somatostatin receptor antagonists, a paradigm shift for more effective PRRT?

#### By Frederik Cleeren

Peptide receptor radionuclide therapy (PRRT) using radiolabeled somatostatin (SST) analogues has been employed since the early 1990s to treat somatostatin receptor (SSTR)-positive neuroendocrine neoplasms (NENs). Initially, cell-internalizing SSTR agonists were used as vector molecule for PRRT of NENs. In 2006, the concept of SSTR antagonists was introduced for targeting NENs (Ginj et al. 2006). Both preclinical and clinical studies demonstrated much higher tumor accumulation of these non-internalizing SST analogues than for SSTR agonists.

Over the last decade, terbium-161 has gained increasing attention as a potential therapeutic radionuclide. It has similar decay characteristics to lutetium-177 but, in addition, terbium-161 co-emits a substantial number of short-ranged electrons (conversion and Auger electrons) characterized by a high linear energy transfer (4–26 keV/µm). It was commonly believed that nuclear localization is essential to induce DNA-double strand breaks by short-ranged electrons, resulting in therapeutic effects. However, it has recently been shown that the cell membrane may also be a suitable target for Auger electron emitters (Paillas et al. 2016).

**Fig. 2:**
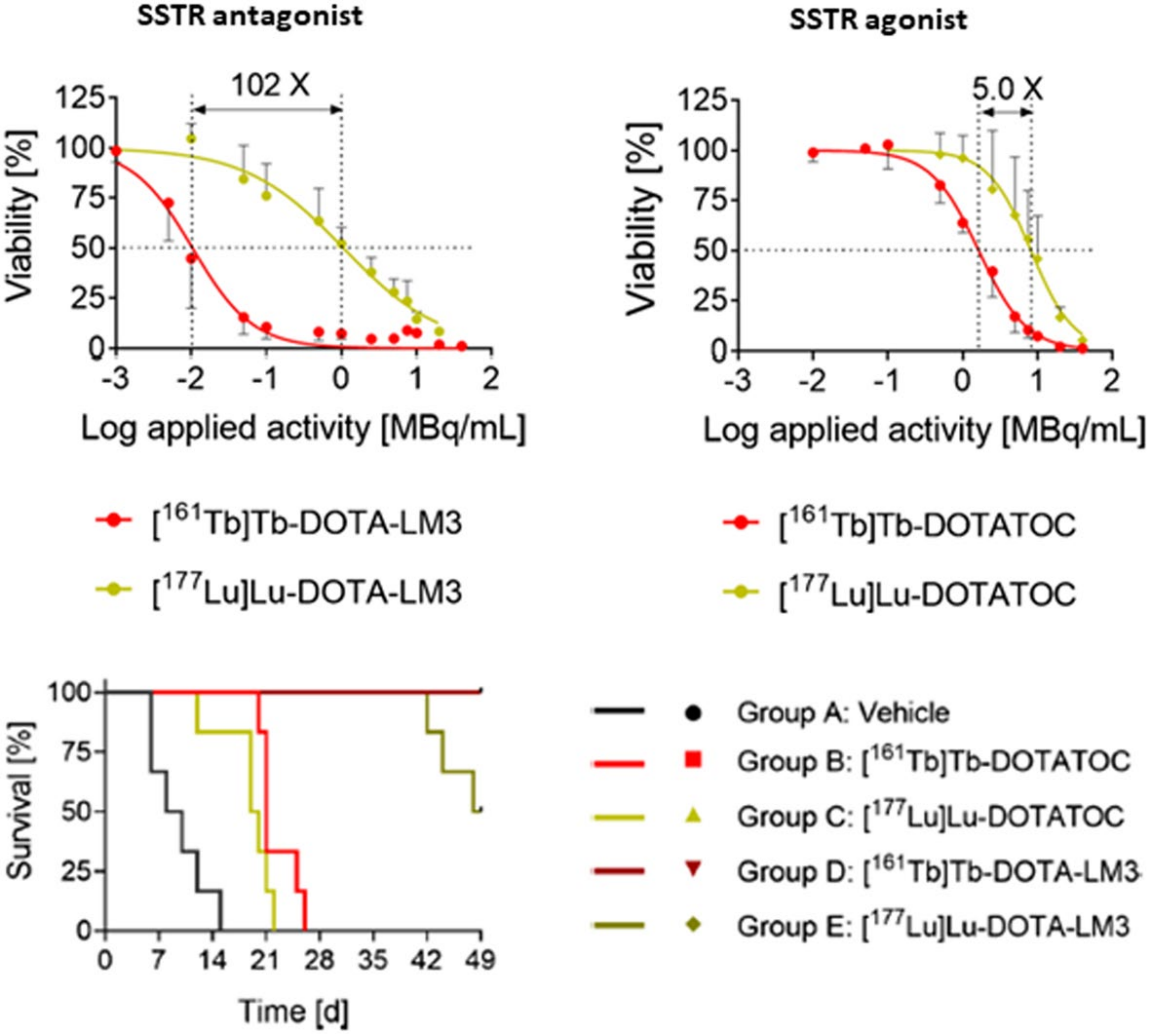
AR42J tumor cell viability (MTT assay) and Kaplan–Meier plot (2 × 10 MBq; 0.2 nmol) for ^161^Tb- and ^177^Lu-labeled SSTR agonists (DOTATOC) and antagonists (DOTA-LM3) in AR42J tumor-bearing mice. (adapted from Borgna et al. 2022)

Borgna et al. have shown indeed that the non-internalizing SST antagonist [^161^Tb]Tb-DOTA-LM3 was 102-fold more potent than [^177^Lu]Lu-DOTA-LM3 as can be seen in Fig. 2 (Borgna et al. 2022). Although, the ^161^Tb-labeled SST agonist DOTATOC was only fivefold more effective inhibiting tumor cell viability than its ^177^Lu-counterpart. This result was confirmed in vivo and demonstrated that the membrane localizing [^161^Tb]Tb-DOTA-LM3 was significantly more effective in delaying tumor growth than both [^177^Lu]Lu-DOTA-LM3 and [^161^Tb]Tb-DOTATOC. [^161^Tb]Tb-DOTA-LM3 is thus a promising clinical candidate for PRRT, combining the advantages of SSTR antagonists with the advantages of terbium-161. This might initiate a paradigm shift towards the application of Auger-electron emitters targeting the cell membrane instead of nuclear DNA.

### A simple and automated method for ^161^Tb purification and ICP-MS analysis of ^161^Tb

#### By Jonathan Engle

It is increasingly accepted that ^161^Tb has therapeutic advantages compared to ^177^Lu (*vide supra*), as well as similar production potential. A recent article by McNeil recognizes a confounding limitation of ^161^ Tb production, which has been spearheaded by groups at the Paul Scherrer Institute in Switzerland for the past several years, namely, that the highest quality productions of ^161^Tb reported so far are constrained by the availability of uniquely small meshed ion exchange resin materials (McNeil et al. 2022). While other high performance ion chromatography- (HPIC-) based separation methods are being developed elsewhere, high-pressure processes present particular challenges to scale-up and are substantially more challenging to implement than the cartridge-based method reported here using materials that are readily commercially available at reasonable cost. Wider availability of the ^161^Tb radionuclide will enable comparative evaluation against ^177^Lu which is needed to hone clinical paradigms whose regulatory processes require many years of effort to realize, and to ask more fundamental dosimetric questions about the role that emitted electron energy distribution plays in therapeutic efficacy.

### [^18^F]fluoride on TLC: expecting the unexpected!

#### By Marianne Patt

Already since the very beginnings of PET radiochemistry using [^18^F]fluoride in the 1980s, determination of radiochemical purity by HPLC has been supplemented by an additional TLC method in order to circumvent underestimation of free unreacted starting material (i.e. [^18^F]fluoride) in the final radiopharmaceutical preparation. Compared to today, the HPLC columns that were available in the early days were manufactured using less pure bed materials and often free non-carrier-added [^18^F]fluoride simply did not elute from the chromatographic column. This resulted in estimation of an artificially high radiochemical purity due to the apparent complete absence of unreacted starting material. Nowadays, the HPLC columns that are supplied by commercial chromatography companies are manufactured from much purer high quality stationary phases and, in most cases, do no longer suffer from incomplete [^18^F]fluoride recovery. However, the complementary assessment of radiochemical purity by TLC has found entrance into the tests that are prescribed in several monographs of the different pharmacopoeias.

The authors of a technical note found a surprising distribution pattern of free [^18^F]fluoride on silica TLC plates when relatively high amounts of water in the mobile phase mixture were used (Laferriere-Holloway et al. 2023). Although the behavior is explainable by the different types of interactions of the analyte with the stationary phase, the findings are still somewhat unexpected. These results are of considerable importance since the radiochemical purity is an important release parameter for an individual batch of an ^18^F-labelled radiopharmaceutical, and qualified persons responsible for batch release should be made aware of the possible influence of mobile phase mixture on analysis. Not unexpectedly this work highights that thorough validation of an analytical procedure is required in order to obtain scientifically sound results, and is an example in radiopharmacy where GMP-requirements prove their right to exist.

### Depopulating the graveyard of failed PET radiopharmaceuticals with total body PET

#### By Peter J. H. Scott

Early in my faculty career, I was in a dour mood after receiving a less than favorable grant review. Mike Kilbourn noticed my drab outlook, handed me an old typewritten document and said “this will make you feel better. It’s the review of the first grant I wrote on carbon-11 chemistry in the 1980s”. Kilbourn’s grant also received the dreaded “not discussed”, which represents undisputable death for proposals submitted to NIH, and review highlights included: *poor radionuclide choice*, *20 min half-life too short*, and *radiochemistry with* ^*11*^*C is not feasible*.

While there are limitations to working with ^11^C, history tells us the reviewers were wrong. Articles highlighted in this Editorial include a review showcasing decades of imaging with ^11^C-labeled molecules (Shegani et al. 2023), and offer a valuable lesson to young scientists—don’t give up on your ideas just because reviewers cannot see the possibilities!

Another new technology that has had an uphill battle against reviewers is total body PET (TBP) pioneered by Simon Cherry. Imaging applications of TBP have been widely discussed elsewhere but, in a recent article, de Vries and colleagues consider what the technology means for radiochemists (de Vries et al. 2022), and ask whether the power of these new scanners (e.g. increased imaging quality, lower dose/molar activity (A_m_) requirements, increased sensitivity and higher signal to noise ratios) can *depopulate the graveyard of failed PET radiopharmaceuticals*?

Coming full circle, the authors note benefits of TBP are particularly attractive when working with ^11^ C. Lower dose and A_m_ requirements offer potential for repurposing synthesis methods considered too low yielding in the past, as well as resurrecting legacy radiotracers that historically could not be prepared in sufficient amounts for use with traditional scanners. The ability to inject less activity and perform quicker scans also enables injection of multiple patients from single radiotracer batches, and distribution to satellite imaging centers without a cyclotron. New possibilities include later scans for tracers with slow kinetics, currently not practical with traditional scanners. Although TBP scanners remain expensive, costs are expected to drop which, in combination with the benefits and ability to scan more patients per day, will make the economics more manageable. TBP technology should become more commonplace, presenting exciting new possibilities for radiochemistry development in the future.

### Do antibodies have a place in the strategies of cancer treatment using internal vectorised radiotherapy (IVR)?

#### By Alain Faivre Chauvet

A complete validation of an anti-CD123 antibody is described (Laszlo et al. 2022) from its production to use in a mouse model, and offers a very interesting discussion on the place of the radiolabelled antibody in the treatment of acute leukemia. With the start of clinical trials for the treatment of endocrine tumours and prostate cancer, the role of peptides radiolabelled with alpha-emitters such as [^225^Ac]actinium is becoming better defined. However, that of antibodies, which are characterised by particular pharmacokinetics, remains to be done. To this end, the use of an [^211^At]astate radiolabelled antibody to effectively treat a mouse model of acute leukaemia with RIV-Alpha, with acceptable side effects, is a step forward for the field (Laszlo et al. 2022). The originality of this article also lies in the fact that it describes the possible use of IVR-Alpha with an innovative radionuclide, [^211^At]astate, which is not subject to the current problems of access to radionuclides produced by neutron irradiation or of the nuclear waste reprocessing chain. It also demonstrates that, provided that a therapeutic target is chosen that is easily accessible by the bloodstream and is poorly or not at all expressed by the surrounding healthy tissues, the use of antibodies should be considered in IVR-Alpha treatments in view of their long tumour residence time. Indeed, their plasma half-life and tumour residence time, which are generally longer than those of peptides, result in favourable tumour dosimetry, making these vectors candidates for consideration in IVR-Alpha.

### ^18^F-Pretomanid PET imaging from animal to clinical

#### By Hua Zhu

Infections like tuberculosis (TB) remain one of most common diseases threatening human development. Multidrug-resistant (MDR)-TB, caused by mycobacterium tuberculosis resistant to first-line antibiotics is on the rise. New drugs and more efficacious treatments against MDR-TB are therefore urgently needed. However, essential information about drug concentrations reaching the site of infection is still missing. Sanjay K. Jain and colleagues developed and synthesized ^18^F-pretomanid as a PET-radiotracer to noninvasively assess whole-body drug biodistribution (Mota et al. 2022).

Radiosynthesis and quality control of ^18^F-pretomanid was performed under current Good Manufacturing Practice (cGMP). Micro-PET studies in mouse and rabbit models of TB meningitis were conducted. First-in-human dynamic ^18^F-pretomanid PET studies were also performed, in accordance with local government guidelines. This research showed the value added by imaging technologies for the characterization of novel drugs and treatment optimization in infectious diseases. Considering the reliable and convenient labeling scheme, ^18^F-pretomanid shows great potential for further clinical applications around infection imaging.

### Novel macrocyclic chelator for alpha-emitter complexation with high stability

#### By Zhen Cheng

The field of radionuclide therapy is rapidly emerging and is at the forefront of research in nuclear medicine. Especially, alpha radionuclide-based therapy has shown great promise for realizing desirable cancer treatment outcomes. Therefore, it is of great significance to design and develop novel effective chelators for complexation of alpha-emitters for targeted therapy of tumor, such as ^225^Ac. Though numerous research efforts have been devoted to the search for such molecules, currently available ^225^Ac chelators still suffer from a variety of problems, especially relatively low stability for biomedical applications.

**Fig. 3:**
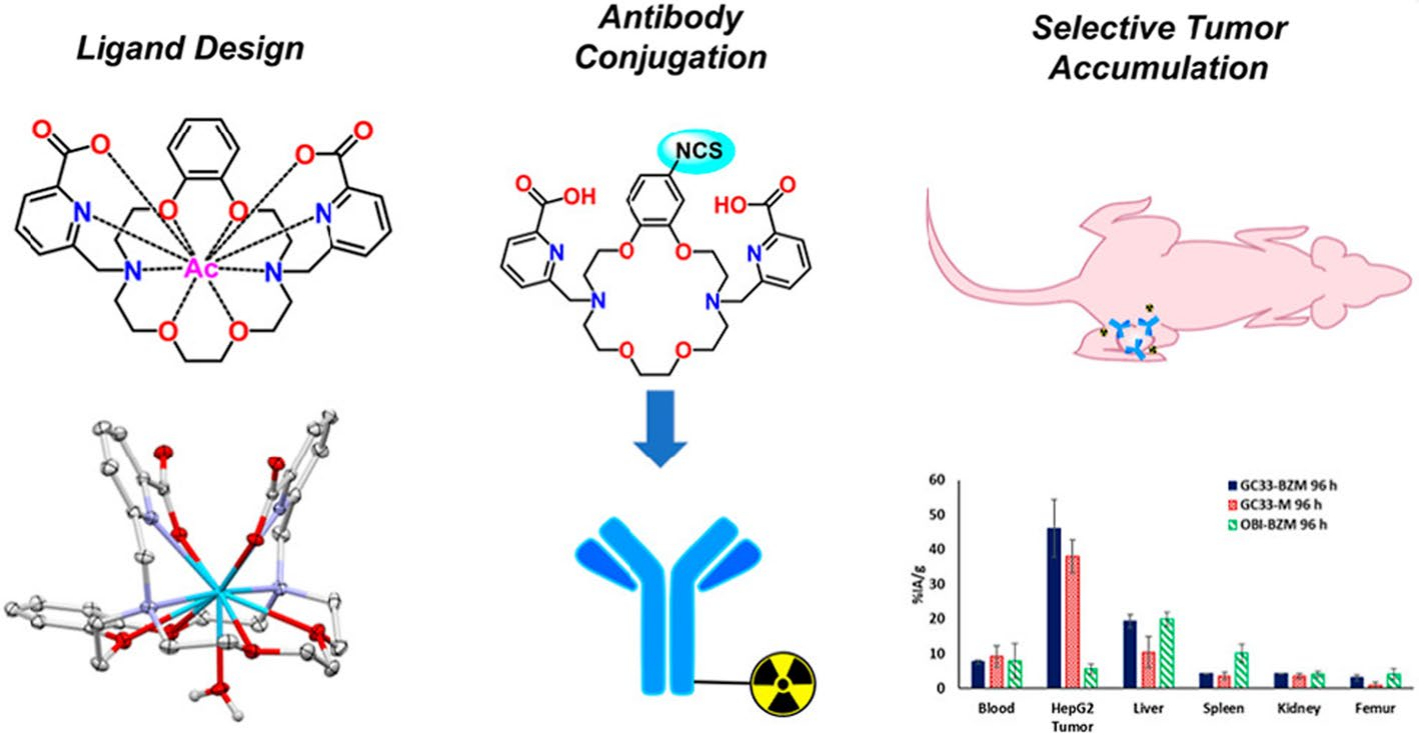
Overview of use of new H2BZmacropa chelator, its conjugation to an antibody and biodistribution results. (from Bioconjugate Chem. 2022;33, 6, 1222–1231)

A new publication elegantly applied ligand design principles to obtain a novel bifunctional chelator for ^225^Ac, H2BZmacropa-NCS (Kadassery et al. 2022). Previously, the researchers demonstrated the usefulness of the H2macropa molecule scaffold for ^225^Ac-chelation (Fig. 3). Through equipping a benzene ring directly on the macrocycle in this work, the design reduced the conformational flexibility of the ligand and facilitated the molecule to present in a preorganized form for high efficiency and rapid chelation of Ac^3+^ under mild conditions. Moreover, installation of an NCS group on the benzene ring in the backbone of the macrocycle improved the hydrolytic stability of the resulting ligand, which broadens the availability of the compound through easy shipping and storage. The H2BZmacropa-NCS was then conjugated with GPC3 targeted MAb GC33 and radiolabeled with ^225^Ac to obtain the alpha-targeted radionuclide therapy agent, ^225^Ac-GC33-BZM. Further evaluation of the radiolabeled mAb indicated that the serum and in vivo stability of the agent was acceptable but could be further optimized for clinical development. H2BZmacropa-NCS demonstrates high promise for modification of biomolecules and radiolabeled with alpha-emitters for targeted treatment.

### Potential imaging of collageninduced arthritis using ^64^CuimmunoPET

#### By Amir R Jalilian

Copper-64 provides characteristics of a suitable radioisotope for imaging physiological processes within hours-days’ time span. Though not a perfect match for full-size antibodies, the half-life of ^64^Cu (12.7 h) nicely matches the biological half-life of fragments such as F(ab)’_2_s. Several worldwide concerted activities covered different aspects of copper-64 radiopharmaceuticals used in PET diagnosis of various diseases, with published documents including [^64^Cu]-ImmunoPET (IAEA, 2022). Inflammation nuclear imaging, including autoimmune rheumatoid arthritis (RA), is still not a well addressed area and CD4+ T cell imaging might be a nice approach to image RA, using immuno-PET agents (Kinne et al. 2010). In a new study, the tracer, [^64^Cu]Cu-NOTA-CD4-F(ab)’_2_, a NOTA-based radiolabeled immunomolecule based on anti-mouse CD4 antibodies, was developed and evaluated using classic preclinical tests including flow cytometry, immunohistochemistry, biodistribution studies in a collagen-induced arthritis (CIA) mice model (with or without corticosteroid treatment). The results suggest [^64^Cu]Cu-NOTA-CD4-F(ab)’_2_ is a novel CD4 PET tracer for use in non-invasive visualization of murine CD4+ T cells (Skovsbo Clausen et al. 2022). The small cavity size of the NOTA chelate (Takahashi et al. 1977) can cause a distorted geometry around Cu^2+^, resulting in limited complex stability. Further studies with more stable copper chelates (such as sarcophagines) can possibly increase the image quality. On the other hand, high kidney background should be also considered before possible future human trials. Overall, the work provides a nice approach for RA imaging.

### But what cell type is my tracer targeting ?

#### By Benjamin Guillet

Combining in vivo radiotracer exposure, cell sorting with magnetic antibodies, flow cytometry and gamma counting, Laura Bartos and colleagues developed and validated a method allowing isolation of tumor and immune cells from a resected tumor, and counting their respective radioactivity content (Bartos et al. 2022). This allows the team to evaluate radiotracer uptake according to cell types in heterogenous tissue.

Applied in vivo to a glioblastoma model in mice, and ex vivo in human glioblastoma tissue, using a TSPO radiotracer, they surprisingly observed that TSPO uptake was more intense in tumor cells than in immune cells. Applied to human tissue, a similar picture together in low and high grade Glioblastoma patients was found, and moreover, they observed significantly higher TSPO radiotracer uptake of single tumor cells in high grade patients compared to low grade.

This really innovative and helpful method opens the door to a better understanding of PET and SPECT images obtained by molecular imaging.

### A how-to guide for radiosynthesis automation

#### By Michael van Dam

Commercial cassette-based radiosynthesis modules are becoming increasingly used for automation of radiopharmaceutical production because the use of a disposable fluid path greatly simplifies operation and compliance with cGMP requirements. Currently, only a limited number of radiopharmaceuticals are officially supported by manufacturers in the form of pre-defined kits and computer scripts, and there remains a high need and interest for end users to develop their own automated protocols, especially in the case of clinical translation of novel tracers. Unfortunately, implementation of reliable and efficient synthesis protocols on such platforms can be challenging due to the significant amount of background knowledge required, for which there is a shortage of structured educational resources.

A recent tutorial article by Barnes makes great strides toward filling this knowledge gap (Barnes et al. 2022). This tutorial provides a very accessible and well-illustrated introduction to the architecture and components of cassette-based synthesis modules, and to general strategies for implementing a radiosynthesis on such modules (including cassette design and process automation). At the end of the article is a brief case study on the synthesis of [^18^F]TTCO-IL2. Furthermore, the authors describe how to mitigate many potential pitfalls that may not be immediately apparent to newcomers to the field, including dead volumes in reagent vials, cross-contamination of fluid pathways, and the need for well-controlled fluoride drying conditions.

### How [^18^F]AlF is phagocyting the clinical ^68^Ga applications

#### By Johnny Vercouillie

**Fig. 4:**
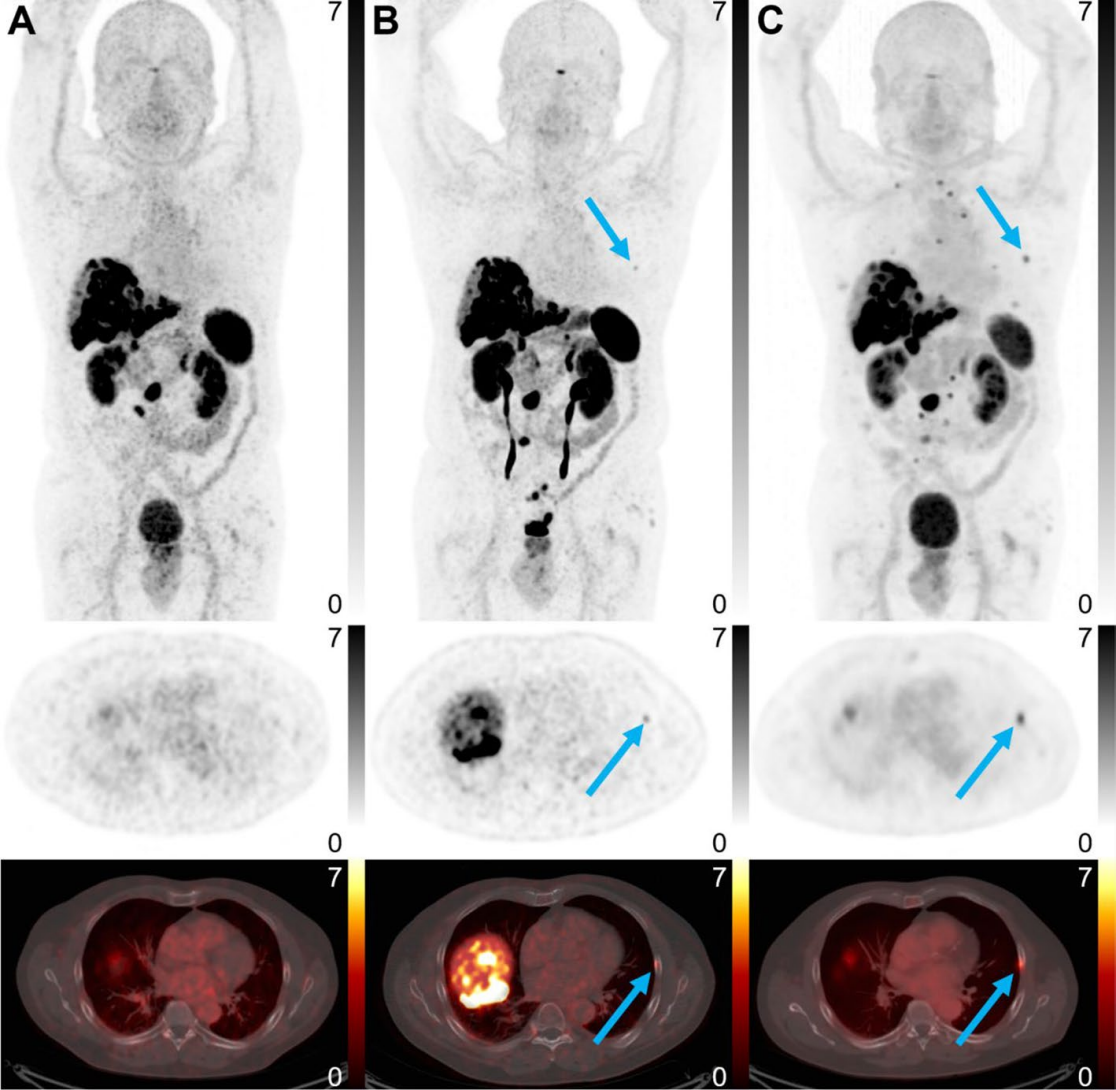
**A** ^68^Ga-DOTANOC and **B** ^18^ F-AlF-OC images (from top to bottom: maximum-intensity projection PET, transversal PET and fused PET/CT images, respectively) of a seventy-year old male patient with an intestinal neuroendocrine tumor with multiple liver metastases and one involved lymph node. ^18^ F-AlF-OC detected an additional bone lesion (blue arrow) that was confirmed by **C** follow-up ^68^Ga-DOTANOC imaging 6 months later. Lookup tables apply to PET images (SUV). (From Pauwels et al. 2022)

In a prospective trial (NCT03883776) the results of injection of [^68^Ga]Ga-DOTATATE and [^18^F]AlF-NOTA-octreotide in six healthy volunteers, and [^18^ F]AlF-NOTA-octreotide in six NET patients who already underwent the [^68^Ga]Ga-DOTATATE PET in the previous 6 months have been reported (Pauwels et al. 2020). As the results were very encouraging, a larger study was required to demonstrate the non-inferiority of [^18^F]AlF-NOTA-octreotide compared to [^68^Ga]Ga-DOTATATE. Thus, seventy-five patients with histologically confirmed NET and a routine clinical ^68^Ga-DOTATATE (n = 56) or ^68^Ga-DOTANOC (n = 19) PET were enrolled in a clinical trial (NCT04552847) to evaluate [^18^F]AlF-NOTA-octreotide performance (Pauwels et al. 2022). The non-inferiority trial demonstrated that among all tumor lesions detected by the three different radiopharmaceuticals, the [^18^F]AlF-NOTA-octreotide presented a significantly higher detection ratio compared to both ^68^Ga-radiopharmaceuticals. Moreover, the tumor-to-background ratio was significantly more favorable for [^18^F]AlF-NOTA-octreotide (Fig. 4). Finally, as reported by the authors, [^18^F]AlF-NOTA-octreotide appears to be superior to [^68^Ga]Ga-DOTATATE. Further comparisons of radiopharmaceuticals either labelled with [^18^F]AlF or ^68^Ga, as already initiated as for FAPI-74 (Giesel et al. 2021) and PSMA-11 (De Man et al. 2022), should rapidly be able to decide between both radionuclides for routine applications. Nevertheless, outside clinical considerations, a wider use of [^18^F]AlF instead of ^68^Ga-labeled radiopharmaceuticals should benefit patients and healthcare professionals. A centralized large-scale production of [^18^F]AlF radiopharmaceuticals allowed by the half-life of ^18^F will drastically reduce the radiation exposure of staff in radiopharmacies, increase availability of these radiopharmaceuticals for patient care and reduce the costs of their production.

### Combination of targeted radionuclide therapy and immunotherapy is beneficial but timing is of high importance

#### By Martin Behe

Minnix and Kujawski et al. investigated the impact of a combination of cytokine immunotherapy and a radiolabeled ^225^Ac-labeled antibody (M5A) targeting carcinoembryonic antigen (CEA) (Minnix et al. 2022). They used a syngeneic orthotopic breast carcinoma mouse model in immunocompetent mice for this purpose. They observed about a 20-day delay in tumor growth when CEA-positive mammary tumors were treated with 7.4 kBq ^225^Ac-M5A as well as with single immunocytokine treatment. When the two treatments were combined, no significant improvement over monotherapies was observed when targeted alpha therapy (TAT) with 7.4 kBq ^225^Ac-M5A was administered 5 days after immunotherapy, while immunotherapy 10 days after TAT resulted in a higher delay in tumor growth of 38 days!

The authors also examined immune cell infiltration by immunophenotyping and immunohistochemistry (IHC). They observed significantly decreased infiltration of both CD4 and CD8 positive T cells at the highest dose of TAT, whereas a nonsignificant increase was observed with immunocytokine treatment. They conclude that pretreatment with immunocytokines may interfere with an increase in efficacy of TAT that could kill the infiltrated T cells. This article shows that combining immunotherapy with targeted radionuclide therapy is beneficial, but that timing is crucial.

### Preclinical evaluation of ^99m^Tc labelled EGFR imaging agents: potential contribution to a better management of cancer patients

#### By Ana Rey

In the search of novel radiopharmaceuticals to broaden the spectrum of diagnostic and therapeutic options in the fight against cancer, a series of ^99m^Tc complexes directed against the Epidermal Growth Factor Receptor (EGFR) was presented (Kiritsis et al. 2022). This cell-surface receptor is overexpressed in a wide variety of cancers such as head and neck, ovary, breast, colon, and lung cancer, and frequently seems to confer an adverse prognosis. Furthermore, EGFR inhibitors are successfully used in the chemotherapy of EGFR positive tumors.

**Fig. 5:**
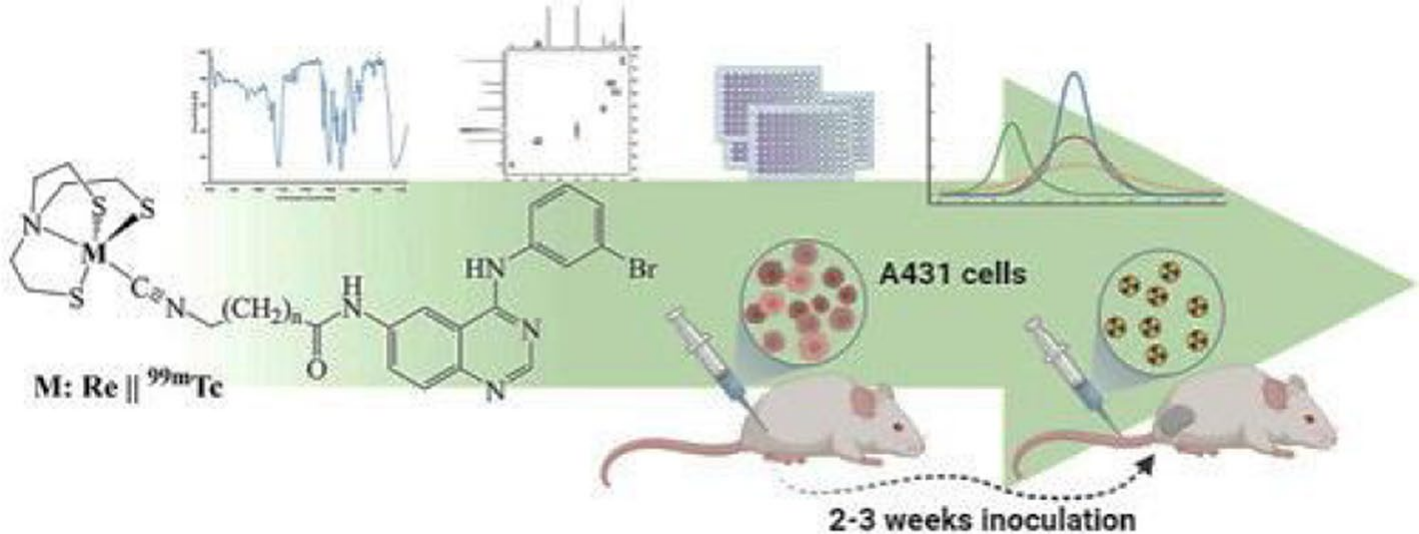
Schematic overview of preclinical experiments to evaluate ^99m^Tc labelled EGFR imaging agents. (from Kiritsis et al. 2022)

In this paper, the authors propose to develop ^99m^Tc-labeled 4-aniloquinazoline radiotracers to accurately and reliably predict the sensitivity of patients to anti-EGFR treatments. From the various possible technetium labeling approaches they select the use of “4 + 1” mixed-ligand system [M(NS3)(CN-R)], in which the biomolecule derivatized with an isocyanide acts as a monodentate ligand and the tripodal chelator 2,2′,2″-nitrilotris(ethanethiol) acts as the tetradentate coligand. These types of complexes are flexible, robust and stable, and constitute a good alternative for labeling small biomolecules without loss of their activity. Besides the synthesis of the ligands, the labeling and the structural elucidation by using stable rhenium analogs, the paper includes a full set of physicochemical studies, in vitro screening for their potency to inhibit the growth and ability to inhibit EGFR phosphorylation in A431 cells (Fig. 5). Interestingly, this study also describes the first evaluation of a ^99m^Tc aniloquinazoline complex in a tumor bearing mouse model.

In a moment in which advancements in solid-state detector technology are fueling a rediscovery of technetium-99 m, the development of this type of study contributes to a better management of cancer patients.

### Towards a new wave of ^18^F-Labeled Tracers

#### By Silvio Aime

**Fig. 6:**
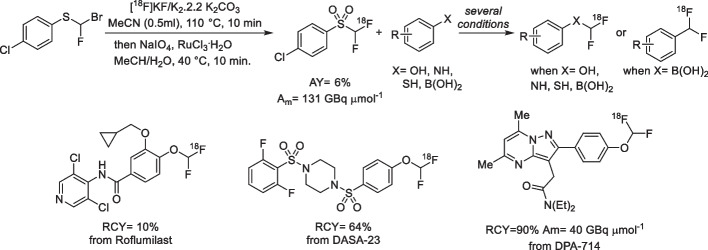
Schematic overview of [^18^F]difluorocarbene precursors obtained through a halogen exchange ^18^F/Br of (bromofluoromethyl)(4-chlorophenyl)sulfane starting material, followed by an oxidation step. Examples of produced ^18^F-radioligands are presented

Positron emission tomography has broadened the range of research and clinical applications of radionuclides, although, new challenges have arisen such as the development of novel and diverse radiolabelling methodologies. Those issues have accelerated the progress in fluorine-18 radiochemistry with numerous methods available to ^18^F-label (hetero)arenes and alkanes. Many compounds not only possess a fluorine atom in their scaffold but also, sometimes, bear a polyfluoroalkylated chain, those features are perfect to expand the chemical space of radiopharmaceuticals. However, to date, the ^18^F-labelling of polyfluoroalkylated molecules mostly remain an unsolved problem mainly due to their low molar activity (A_m_) when radiolabelled, a considerable drawback considering the omnipresence of these motifs in drug discovery. The Gouverneur group developed an elegant solution towards the labelling of difluoromethylated molecules through a new [^18^F]difluorocarbene reagent, [^18^F]1-chloro-4-((difluoromethyl)sulfonyl)benzene (Sap et al. 2022). This difluorocarbene precursor is obtained through a halogen exchange ^18^F/Br of (bromofluoromethyl)(4-chlorophenyl)sulfane starting material, followed by an oxidation step (Fig. 6). The issue of A_m_ is solved using an assay examining the likelihood of isotopic dilution on variation of the electronics of the difluorocarbene precursor, revealing Cl as the best group to keep a high A_m_. The versatility is demonstrated with multiple [^18^F]difluorocarbene-based reactions including O–H, S–H and N–H insertions, and cross-couplings that harness the reactivity of ubiquitous functional groups such as (thio)phenols, N-heteroarenes and aryl boronic acids that are easy to install. The combination of high A_m_ and a simple radiofluorination protocol should encourage rapid adoption of this new methodology.

### Imaging leucine-rich repeat kinase 2 in vivo using Positron Emission Tomography (PET)

#### By Jan Passchier

Leucine-rich repeat kinase 2 (LRRK2) is believed to play an important role in the progression of Parkinson’s disease, with significant efforts underway to identify promising inhibitors to test for therapeutic efficacy. The availability of a specific and selective PET ligand would greatly benefit these efforts, allowing for drug-occupancy studies and supporting better disease understanding.

**Fig. 7:**
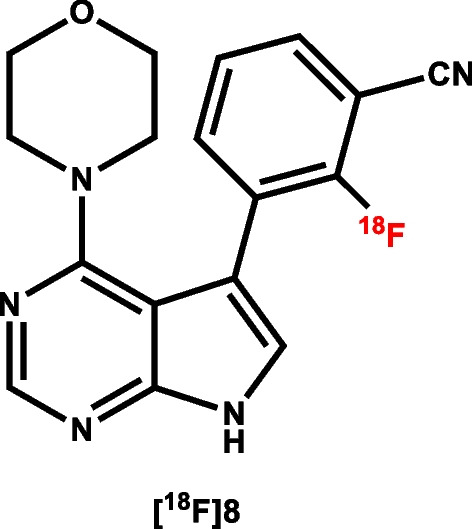
Structure of [^18^F]8

In their recent paper, Chen et al. present a series of promising candidates for further exploration (Chen et al. 2022). Of these, [^18^F]PF-065455943 ([^18^F]8, Fig. 7) demonstrated high potency (IC_50_ = 3.58nM) towards LRRK2 and good in vitro specific binding signal in accordance with anticipated LRRK2 expression. Having appropriate physicochemical properties and good serum stability across species, [^18^F]8 was tested in non-human primates (NHP) to assess its in vivo characteristics. Following administration, [^18^F]8 concentration quickly peaked in the brain followed by a relatively fast washout. Compartmental analysis using the metabolite corrected arterial input function and a 2-tissue compartmental model gave total volumes of distribution (V_T_) ranging from 3.5 to 4.6 mL/cm^3^. Pre-treatment using a structurally related inhibitor led to a reduction in V_T_, indicating specific binding but the observed reduction appeared to be global in nature rather than reflecting LRRK2 expression. While promising, this global reduction in signal following the blocking experiment is cause for some concern with respect to the in vivo selectivity of [^18^F]8 for LRRK2.

Given these results, further studies will be required to confirm if this tracer can deliver on its promise.

### EU GMP Annex 1, a real page-turner?

#### By Lars Perk

Radiopharmaceuticals for parenteral administration should comply with sterility requirements and, where relevant, aseptic working conditions for the manufacture of sterile medicinal products (Eudralex Volume 4, Annex 1).

The revision of EU GMP Annex 1 was finalized in August of 2022 by the European Commission. The deadline for coming into operation is 25 August 2023. The new version of Annex 1 has expanded from 16 to 58 pages(!), and includes new requirements as well as much more detail related to many of the requirements presented in the previous version. It also contains a strong focus on risk management - the term ‘Risk assessment’ is mentioned 20 times - and contamination control strategy (CCS Task Force issues new Guideline).

Some of the new requirements might be difficult or even impossible to implement for a manufacturer of radiopharmaceuticals, for example:

- For isolators (i.e. dispensing hot-cells), the bio-decontamination process of the interior should be automated, validated and controlled within defined cycle parameters and should include a sporicidal agent in a suitable form (e.g. gaseous or vaporized form);
- The integrity of the sterilised filter assembly should be verified by integrity testing before use (pre-use post sterilisation integrity test or PUPSIT (Parental Drug Association));
- The bioburden assay should be performed on each batch for both aseptically filled products and terminally sterilised products and the results considered as part of the final batch review.

Besides the above requirements, specific requirements for single use systems (e.g. dispensing sets or cassettes) were also introduced. Overall, the new Annex 1 requires careful reading and a thorough risk assessment.

### The hitchhiker’s guide to non-clinical studies with radiopharmaceuticals

#### By Francisco Alves

This document is the result of a Technical Meeting of the International Atomic Energy Agency (IAEA) held in Coimbra in November 2021, with the aim of establishing a consensus paper on the non-clinical studies for the clinical translation of radiopharmaceuticals (Korde et al. 2022).

It is aimed as a guide for radiopharmaceutical scientists, Nuclear Medicine specialists, and regulatory professionals to bring innovative diagnostic and therapeutic radiopharmaceuticals into the clinical evaluation process in a safe and effective way.

The document covers the main regulatory requirements including non-clinical pharmacology, radiation exposure and effects, toxicological studies, pharmacokinetic modelling, and imaging studies. Additionally, standardization of different specific clinical applications is also discussed.

The document is a great tool for anybody pursuing translation of radiopharmaceuticals from the preclinical stage into a clinical trial, and constitutes a great guide to help navigating the complex regulatory landscape that regulates this field.

## Conclusions

Trends in radiochemistry and radiopharmacy are highlighted demonstrating the progress in the research field being the scope of EJNMMI Radiopharmacy and Chemistry.

## Data Availability

Datasets mentioned in this article can be found in the cited articles.

## Abbreviations

Ab: Antibody
BBB: Blood brain barrier
Bq: Becquerel
DFO: Desferrioxamine
DNA: Desoxyribo nucleic acid
DOTA: 2,2′,2′′,2′′′-(1,4,7,10-Tetraazacyclododecane-1,4,7,10-tetrayl)tetraacetic acid
EGFR: Epidermal growth factor receptor
GMP: Good manufacturing Practice
IAEA: International atomic energy agency
IHC: Immuno histochemistry
IVR: Internal vectorized radiotherapy
LRRK2: Leucine-rich repeat kinase 2
MDR: Multidrug resistance
NEN: Neuroendocrine neoplasms
NOTA: 1,4,7-triazacyclononane-1,4,7-triacetic acid
PET: Positron emission tomography
PRRT: Peptide receptor radionuclide therapy
SPECT: Single photon emission computed tomography
SST: Somatostatin
TB: Tuberculosis
TBP: Total body PET
TFP: Tetrafluorophenyl
